# Supplementation of Sulfur-Containing Amino Acids or Essential Amino Acids Does Not Reverse the Hepatic Lipid-Lowering Effect of a Protein-Rich Insect Meal in Obese Zucker Rats

**DOI:** 10.3390/nu12040987

**Published:** 2020-04-02

**Authors:** Sandra Meyer, Lea Schäfer, Julia Röhrig, Garima Maheshwari, Erika Most, Holger Zorn, Robert Ringseis, Klaus Eder, Denise K. Gessner

**Affiliations:** 1 Institute of Animal Nutrition and Nutrition Physiology, Justus Liebig University Giessen, Heinrich-Buff-Ring 26-32, 35392 Giessen, Germany; sandra.meyer@ernaehrung.uni-giessen.de (S.M.); lea.schaefer@agrar.uni-giessen.de (L.S.); Julia.Roehrig@agrar.uni-giessen.de (J.R.); Garima.Maheshwari@lcb.chemie.uni-giessen.de (G.M.); erika.most@ernaehrung.uni-giessen.de (E.M.); Klaus.Eder@ernaehrung.uni-giessen.de (K.E.); denise.gessner@ernaehrung.uni-giessen.de (D.K.G.); 2 Institute of Food Chemistry and Food Biotechnology, Justus Liebig University Giessen, Heinrich-Buff-Ring 17, 35392 Giessen, Germany; holger.zorn@lcb.chemie.uni-giessen.de; 3 Fraunhofer Institute for Molecular Biology and Applied Ecology, Winchester Str. 2, 35394 Giessen, Germany

**Keywords:** cysteine, insect meal, liver lipid metabolism, methionine, obese rat

## Abstract

The present study tested the hypothesis that the liver lipid-lowering effect of insect meal (IM) is caused by its low methionine concentration. A total of fifty, male obese Zucker rats were randomly assigned to five groups of 10 rats each (casein (C), IM, IM + Met, IM + Cys, and IM + EAA). While group C received a diet with casein, the IM-fed groups received a diet with IM as the protein source. In groups IM + Met, IM + Cys and IM + EAA, the diets were additionally supplemented with methionine, cysteine and essential amino acids (EAA), respectively. Hepatic concentrations of triacylglycerols and cholesterol, and hepatic mRNA levels and activities of lipogenic and cholesterogenic enzymes were markedly lower in the IM-fed groups than in group C (*p* < 0.05). All of these parameters either did not differ across the IM-fed groups or were only slightly higher in groups IM + Met, IM + Cys and IM+EAA than in the group IM. In conclusion, the results indicate that a difference in the amino acid composition between IM and casein, a low concentration of methionine in IM and a reduced cysteine synthesis secondary to a decreased methionine availability resulting from feeding IM are not causative for the lipid-lowering effect of IM.

## 1. Introduction

An increasing challenge in the world is to provide human population with sufficient amounts of high-quality dietary protein as a source of essential amino acids (EAA). This is mainly because the world population is rapidly growing and, on the other hand, natural resources such as arable land and water, required to produce conventional protein sources (e.g., crops, animal products), are becoming increasingly limited [1]. Thus, alternative protein sources produced in an efficient and sustainable manner are required to meet global demand for dietary protein [2]. In recent years, insect meal (IM) has been recognized as one of such alternative protein sources, because it can be efficiently produced from the large-scale industrialized mass-rearing of edible insects with relatively low environmental impact [3,4]. The potential suitability of IM as a dietary protein source in human nutrition is evident from several recent studies in growing pigs, a well-accepted model organism for humans, showing that feeding IM instead of conventional protein sources neither impairs growth nor causes adverse effects [5,6,7].

Apart from providing EAA and other proteinogenic amino acids for endogenous protein synthesis, various proteins from legume seeds (soybeans, lupins and peas) and potatoes have been shown to exert pronounced metabolic effects, such as liver and plasma lipid-lowering effects compared to casein [8,9,10,11]. Different factors including biologically active peptide sequences [12], specific features of the amino acid pattern (e.g., low methionine content) [8,9] and accompanying substances [13] have been proposed to be causative for these metabolic effects of specific dietary proteins. Interestingly, we have recently demonstrated in two studies with obese Zucker rats that feeding IM from Yellow mealworm (*Tenebrio molitor* L.) instead of casein as a protein source also causes a marked attenuation of liver steatosis and hyperlipidemia due to the inhibition of hepatic triacylglycerol (TG) and cholesterol synthesis [14,15]. However, the responsible factors for the inhibition of hepatic lipid synthesis by IM remain to be identified. One typical characteristic of IM is its low methionine concentration when compared to other animal proteins. Noteworthily, studies in laboratory animals showed that feeding low levels of methionine (“methionine restriction”) causes a strong inhibition of hepatic lipid synthesis and a lowering of blood lipid levels [16,17,18], whereas methionine supplementation causes the opposite and exerts a marked hyperlipidemic action [19]. The effect of methionine on liver lipid synthesis has been attributed to hepatic methionine metabolism, which is linked with glycerolipid metabolism. Hepatic demethylation of methionine provides S-adenosylmethionine (SAM)—a universal methyl donor, which stimulates the methylation of phosphatidylethanolamine into phosphatidylcholine (PC) [20]. PC is subsequently catabolized in the liver to diacylglycerol and subsequently re-esterified to TG [21]. In addition, the demethylation of SAM results in the formation of S-adenosylhomocysteine (SAH), which is further catabolized to homocysteine—a metabolite from methionine catabolism known to stimulate hepatic lipid synthesis [22]. Under conditions of excess availability of methionine, homocysteine is preferentially catabolized to cysteine and glutathione via transsulfuration, whereas under conditions of methionine restriction, the re-methylation of homocysteine into methionine is favored, and methionine is preserved at the expense of cysteine and glutathione [23], and thus the levels of cysteine and glutathione are reduced in methionine-restricted rats [24].

In order to identify the factor responsible for the inhibition of hepatic lipid synthesis by IM, the present study tested the hypothesis that the liver lipid-lowering effect of IM is caused by its low methionine concentration. Thus, an experiment was carried out, in which obese Zucker rats received either a diet with casein or one with an isonitrogenous amount of IM from *Tenebrio molitor* L. as the protein source, and the effects on hepatic lipid concentrations, hepatic lipid synthesis and methionine metabolism were investigated. In order to study whether the lipid-lowering effect of IM is reversed by supplementation with methionine, a third group of rats also received an IM-containing diet, but the dietary methionine concentration was adjusted to that of the group fed casein. Cysteine supplementation was found to reverse the effects of methionine restriction on the expression of genes involved in lipid synthesis [25], which indicates that effects of methionine restriction are not mediated by the decrease of methionine itself, but by a decrease of the downstream amino acid cysteine. Thus, to further study if the lipid-lowering effect of IM involves a lower cysteine synthesis secondary to the low methionine availability, a fourth group of rats received an IM-containing diet with an additional supply of cysteine to reach the level of the sum of methionine and cysteine in the group fed casein. Finally, a fifth group of rats was used, in which the levels of all EAA in the IM diet were adjusted to those of the group fed casein. This group was used to rule out the possibility that the lipid-lowering effect of IM is caused by a generally lower supply of EAA.

## 2. Materials and Methods

### 2.1. Animals and Diets

The animal experiment was approved by the local Animal Care and Use Committee (Regierungspräsidium Giessen; permission no.: JLU 704_M). All experimental procedures described followed established guidelines for the care and handling of laboratory animals. The experiment included 50 male, 10-week-old, homozygous (fa/fa) obese Zucker rats (Crl:ZUC-*Lepr^fa^*) obtained from Charles River (Sulzfeld, Germany). The rats were kept in groups of two animals, each under controlled conditions (12-h light to 12-h dark, 22 ± 1 °C ambient temperature and 50%–60% relative humidity). The rats were randomly assigned to five groups of 10 rats each (casein (C), IM, IM + Met, IM + Cys, and IM + EAA). Five semisynthetic diets with comparable levels of gross energy and crude nutrients were fed (Table 1).

In group C, the semisynthetic diet contained casein as the only protein source, whereas the semisynthetic diets of the other groups contained an isonitrogenous amount of IM, which was produced from the industrial mass rearing of *Tenebrio molitor* L. (Ynsect, Évry, France). The analyzed concentrations of crude nutrients, fatty acids and amino acids of the IM are shown in Table 2.

Owing to the low concentration of methionine in the IM, the diet of group IM was supplemented with 0.9 g methionine/kg diet to achieve a final concentration of 3.2 g methionine/kg diet. This concentration was based on unpublished preliminary studies with IM-based diets, which showed that a concentration of approximately 3 g methionine/kg diet is necessary to allow the normal growth of 10-week-old obese Zucker rats. The diet of group IM + Met was supplemented with 3.1 g methionine/kg diet in order to adjust the dietary methionine concentration to that of group C. The diet of group IM + Cys was supplemented with 3.3 g cysteine/kg diet and, like that of group IM, with 0.9 g methionine/kg diet. The diet of group IM + EAA was supplemented with all essential amino acids in order to adjust the dietary EAA concentrations to those of group C. In order to adjust the concentrations of crude fat and the main fatty acids across the diet of group C and the IM-containing diets, the diet of group C contained 5% soybean oil and the IM-containing diets 1.92% safflower oil and 0.38% linseed oil. The rats had free access to the experimental diets which were fed for 4 weeks. Water was constantly available throughout the experiment ad libitum from nipple drinkers.

After 4 weeks, the rats were decapitated under CO_2_ anesthesia in the fed state. Blood was collected into heparin-coated polyethylene tubes (AppliChem, Darmstadt, Germany), and plasma was separated from blood by centrifugation (1100× *g*; 10 min) at 4 °C. The liver was excised and washed in ice-cold NaCl solution (0.9%), and several aliquots were snap-frozen in liquid nitrogen. Plasma and liver samples were stored at −80 °C pending analysis.

### 2.2. Analysis of Feed Composition

Determination of the total lipid fatty acid composition of IM, the experimental fats and diets, and the chitin content of IM was carried out as described recently [15]. Determination of the dry matter content, energy content and concentrations of crude nutrients and amino acids in the diets and the IM was carried out according to official methods [26,27].

### 2.3. Determination of Hepatic Lipid Concentrations

Total lipids from frozen liver aliquots were extracted with a mixture of n-hexane and isopropanol (3:2, vol/vol), total lipid extracts were dried, and total lipids were dissolved with chloroform and Triton X-100 (1:1, vol/vol) as described recently [15]. Liver TG and cholesterol concentrations were measured using enzymatic reagent kits (Fluitest CHOL, cat. no. 4241; Fluitest TG, cat. no. 5741; both from Analyticon Biotechnologies, Lichtenfels, Germany).

### 2.4. RNA Extraction and qPCR Analysis

Total RNA extraction from frozen liver aliquots (20 mg), cDNA synthesis and qPCR analysis were performed as described recently [15]. The characteristics of the primers used for qPCR analysis are shown in Table S1. The three most stable reference genes used to calculate GeNorm normalization factors according to [28] were: *Actb*, *Canx* and *Mdh1*.

### 2.5. Determination of Hepatic Enzyme Activities

For determination of the hepatic activities of fatty acid synthase (FASN, EC 2.3.1.85) and glucose-6-phosphate dehydrogenase (G6PD, EC 1.1.1.49), liver cytosolic fractions were prepared. In brief, the liver (~100 mg) was homogenized in 1.5 mL 0.1 M phosphate buffer (pH 7.4) on ice with a motor-driven pellet pestle (VWR International, Pennsylvania, USA). The homogenate was centrifuged at 12,000 *g* for 10 min at 4 °C and the supernatant was further centrifuged at 105,000 *g* for 60 min at 4 °C. The final supernatant representing the cytosolic fraction was used for activity measurement. FASN activity was measured by the method of Nepokroeff et al. [29], and G6PD activity was measured using a commercial assay from Sigma-Aldrich (Taufkirchen, Germany; cat. no. MAK015).

### 2.6. Determination of Hepatic Concentrations of Methionine and Its Metabolites

Determination of homocysteine, cysteine and total glutathione in liver homogenates prepared with ice-cold PBS and/or plasma was performed as described recently [15]. Hepatic concentrations of methionine, SAM and SAH were determined by a 3200 QTRAP LC-MS/MS system (Hitachi/VWR, Darmstadt, Germany) according to the method from Burren et al. [30] with slight modifications as described recently [15].

### 2.7. Determination of Plasma Concentrations of Amino Acids

Plasma concentrations of amino acids, with the exception of methionine and tryptophan, were measured according to Dewolfe et al. [31] with modifications. In brief, 100 µL of 10% sulfosalicylic acid was added to 100 µL of plasma to induce protein precipitation. This mixture was incubated for 30 min at 4 °C and centrifuged at 21,100 × *g* for 5 min at 4 °C. A 100 µL-aliquot of the supernatant was diluted with 100 µL of internal standard (0.25 µmol norleucine/mL) and centrifuged again at 21,100 × *g* for 5 min at 4 °C. From the latter supernatant, an aliquot was used to measure amino acid concentrations using an amino acid analyzer (Biochrom 30+, Biochrom, Cambridge, United Kingdom). The plasma concentration of methionine was measured by the method of Burren et al. [30], with modifications, using a 3200 QTRAP LC-MS/MS system (Hitachi/VWR). For determination of the plasma concentration of tryptophan, 50 μL of plasma was mixed with 50 μL of methanol to precipitate proteins. After a 30 min incubation step at 4 °C, the samples were centrifuged at 21,100 × *g* for 5 min at 4 °C. From the supernatant, 25 μL was diluted with 475 μL eluent (water/methanol/phosphonic acid (82.5/17.5/0.1, v/v/v, pH 4)), mixed and measured by a reversed phase-HPLC system (Hitachi/VWR) equipped with a fluorescence detector (L-7485, Hitachi/VWR; excitation wavelength: 283 nm; emission wavelength: 355 nm).

### 2.8. Statistical Analysis

Statistical analyses were performed using the Minitab statistical software (Release 13.1, Minitab Inc., State College, PA, USA). All data were checked for normality of distribution by Anderson-Darling test. Data were analyzed by one-way ANOVA followed by Fisher´s multiple comparison test. Differences were considered significant at *p* < 0.05.

## 3. Results and Discussion

### 3.1. Characterization of the Experimental Diets

As expected, the replacement of casein by IM as a dietary protein source caused a different amino acid composition between the diet of group C and the IM-containing diets (IM, IM + Met, IM + Cys, and IM + EAA; Table 3).

The concentrations of alanine, aspartic acid, glycine and tyrosine were higher and the concentrations of glutamic acid, histidine, isoleucine, leucine, lysine, phenylalanine, proline, serine and threonine were lower in the diets of groups IM, IM + Met and IM + Cys than in the diet of group C. The concentrations of arginine, tryptophan and valine did not differ between the diet of group C and the diets of groups IM, IM + Met and IM + Cys. The concentration of methionine was lower in the diets of groups IM and IM + Cys than in the diet of group C, but was similar between the diets of groups IM + Met and C due to the adjustment of the methionine concentration between these diets. Owing to supplementation of cysteine, the concentration of cysteine was higher in the diet of group IM + Cys than in the diet of group C and in the other IM-containing diets. Due to adjustment of the concentrations of EAA between the diets of group C and group IM + EAA, the concentrations of most EAA (histidine, leucine, lysine, methionine, phenylalanine, threonine and tryptophan) were similar between the diets of these groups, except for isoleucine and valine, which were slightly lower in the diet of group IM + EAA than in the diet of group C. The concentrations of the major fatty acids (linoleic acid, oleic acid, palmitic acid and stearic acid), which contributed to > 90% of total fatty acids, were similar in the diet of group C and the IM-containing diets due to adjustment of fatty acid compositions by using individual amounts of different dietary fats (Table 3). Only the concentration of α-linolenic acid, which was reported to cause hypolipidemic effects in rats [32], slightly differed between the diet of group C and the IM-containing diets. However, since the dietary concentration of α-linolenic acid was even lower in the IM-containing diets than in diet of group C, we exclude the possibility that the α-linolenic acid content is of relevance for the lipid-lowering action of IM. Thus, it is highly unlikely that the metabolic effects induced by the IM-containing diets were confounded by different levels of specific fatty acids between the diet of group C and the IM-containing diets.

### 3.2. The Effect of IM, without and with the Adjustment of Dietary Amino Acids, on the Growth Performance of the Rats

In contrast to our recent study, in which final BWs and daily BW gains were slightly lower in obese Zucker rats fed IM as the protein source instead of casein [15], growth performance parameters (final BW, daily BW gain, feed intake and feed to gain ratio) did not differ between group C and the groups fed the IM-containing diets (Table 4). In addition, relative liver weights did not differ between groups (Table 4).

Most likely, this difference between the present and our recent study is attributed to the fact that the dietary methionine concentration of group IM was slightly higher than in our recent study (3.2 vs. 2.1 g/kg diet) due to supplementation of a small amount of methionine. The dietary concentration of methionine + cysteine in the diet of group IM was 4.6 g/kg diet, which is clearly in excess of the estimated methionine + cysteine requirement for maintenance (2.3 g/kg diet according to National Research Council [33]). The observation that final BWs and daily BW gains were not different across all groups indicates that in the present study, the dietary supply of sulfur-containing amino acids and the other amino acids was also sufficient to allow the adequate growth of the rats and to avoid a growth depression as in our recent study. In line with this, additional supplementation of methionine, cysteine and EAA in the diets of groups IM + Met, IM + Cys and IM + EAA, respectively, did not increase the BW gain of the rats. Thus, these findings suggest that the metabolic effects induced by feeding the IM-containing diets in the present study were not biased by differences in the final BWs and BW gain of the rats.

### 3.3. The Effect of IM, without and with Adjustment of Dietary Amino Acids, on Hepatic Lipid Concentrations and the Expression and Activities of Lipogenic and Cholesterogenic Enzymes

**Figure 1:**
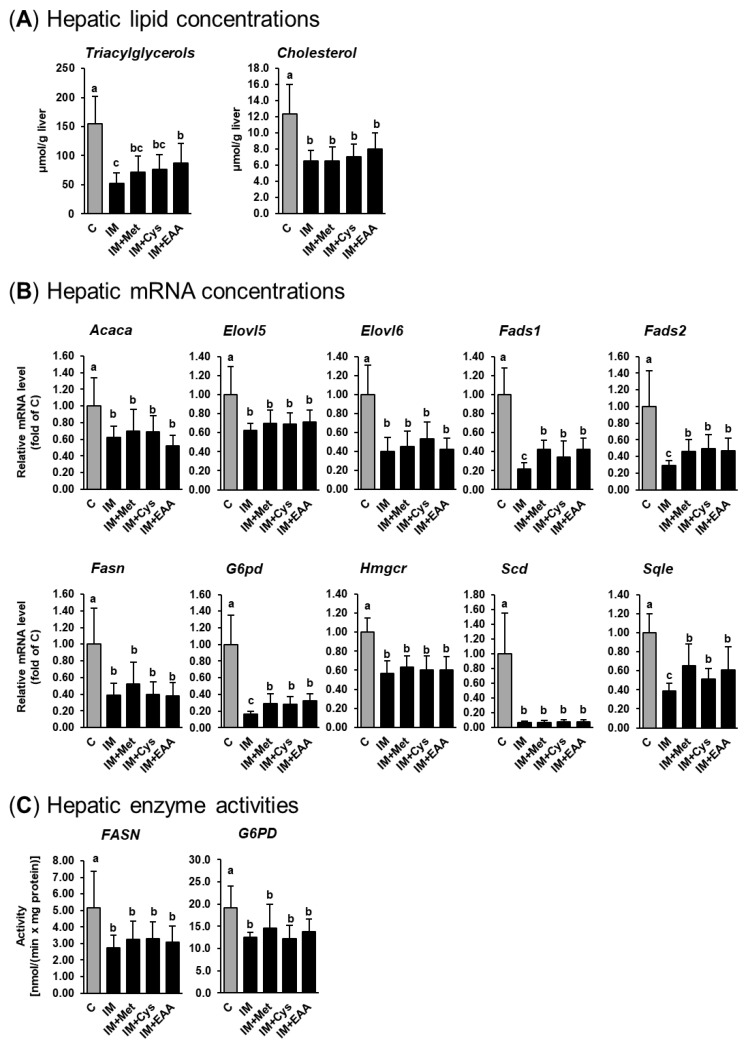
Hepatic TG and cholesterol concentrations (**A**), relative hepatic mRNA levels of lipogenic and cholesterogenic genes (**B**), and hepatic activities of lipogenic enzymes (**C**) in obese Zucker rats fed semi-synthetic diets with either casein (C), insect meal (IM), IM with additional methionine (IM + Met), IM with additional cysteine (IM + Cys) or IM with additional essential amino acids (EAA) for 4 weeks. Bars represent means ± SD for *n* = 10 animals per group. Bars without a common letter differ, *p* < 0.05. Abbreviations: ACACA, acetyl-CoA carboxylase alpha; ELOVL5, fatty acid elongase 5; ELOVL6, fatty acid elongase 6; FADS1, fatty acid desaturase 1; FADS2, fatty acid desaturase 2; FASN, fatty acid synthase; G6PD, glucose-6-phosphate dehydrogenase; HMGCR, 3-hydroxy-3-methylglutaryl-CoA reductase; SCD, stearoyl-CoA desaturase; SQLE, squalene epoxidase.

The present study confirms the observations from our recent studies that IM, compared to casein, causes remarkable liver lipid-lowering effects in obese Zucker rats [14,15]. As shown in Figure 1a, the replacement of casein by IM as the dietary protein source caused a marked reduction of hepatic TG (up to 70%) and cholesterol (up to 50%) concentrations in obese Zucker rats (*p* < 0.05). In contrast to our hypothesis, hepatic concentrations of TG and cholesterol in groups IM + Met and IM + Cys did not differ from those in group IM. This indicates that the low concentration of methionine in the IM and a reduced cysteine synthesis secondary to a lower methionine availability following the ingestion of IM do not account for the liver lipid-lowering effect of IM. While the hepatic concentration of cholesterol did not differ between groups IM + EAA and IM, the hepatic TG concentration was higher in group IM+EAA than in group IM (*p* < 0.05, Figure 1a). These findings indicate that a lower availability of EAA from the IM contributes, at least partially, to the hepatic TG-lowering effect of IM. Nevertheless, the observation that the hepatic TG concentration was still clearly lower in group IM + EAA than in group C suggests that the hepatic lipid-lowering effect of IM cannot be substantially explained by a reduced availability of EAA from the IM.

To address the mechanism underlying the hepatic lipid-lowering effect of IM, the expression and activity of lipogenic and cholesterogenic enzymes in the liver of obese rats were studied. The results of the present study are in line with our recent findings [14,15], that the hepatic lipid-lowering effect of IM is likely mediated by a pronounced inhibition of the expression and activity of lipogenic and/or cholesterogenic genes. As shown in Figure 1b, the mRNA levels of lipogenic (Acaca, Elovl5, Elovl6, Fads1, Fads2, Fasn, G6pd and Scd) and cholesterogenic genes (Hmgcr and Sqle) in the liver were markedly lower (from 40% up to 90%) in group IM and the other IM-fed groups (IM + Met, IM + Cys, and IM + EAA) than in group C. As in our previous studies [14,15], Scd was the most strongly down-regulated gene in the livers of rats fed the IM-containing diets. The mRNA levels of most of these genes (Acaca, Elovl5, Elovl6, Fasn, Hmgcr and Scd) did not differ across the groups fed IM, without or with adjustment of dietary amino acids (groups IM, IM + Met, IM + Cys, and IM + EAA), whereas the mRNA levels of Fads1, Fads2, G6pd and Sqle in the liver were higher in groups IM + Met, IM + Cys and IM + EAA than in group IM (*p* < 0.05, Figure 1b), but were still clearly below those in group C. The hepatic activities of two lipogenic enzymes (FASN and G6PD) were also markedly lower in group IM than in group C (50% and 35%, respectively; *p* < 0.05, Figure 1c) and did not differ between group IM and the other IM-fed groups (groups IM + Met, IM + Cys, and IM + EAA). In the present study, the hepatic activity of the cholesterogenic enzyme HMGCR was not determined, but we have recently demonstrated that IM also inhibits the hepatic activity of HMGCR in obese Zucker rats [14]. Thus, based on the current and our recent findings from gene expression and enzyme activity measurements in the liver, IM causes a profound inhibition of hepatic lipogenesis and cholesterogenesis. Despite the fact that the expression of several lipogenic and cholesterogenic genes was slightly higher in the groups IM + Met, IM + Cys and IM + EAA than in group IM, the expressions of these and all other lipogenic and cholesterogenic genes were still markedly reduced compared to in group C. In line with the effects on hepatic TG and cholesterol concentrations, these results strongly suggest that a reduced availability of sulfur-containing amino acids or EAA from IM does not significantly contribute to the marked lipid-lowering effect of IM.

### 3.4. The Effect of IM, without and with the Adjustment of Dietary Amino Acids, on Hepatic Methionine Metabolism

**Figure 2:**
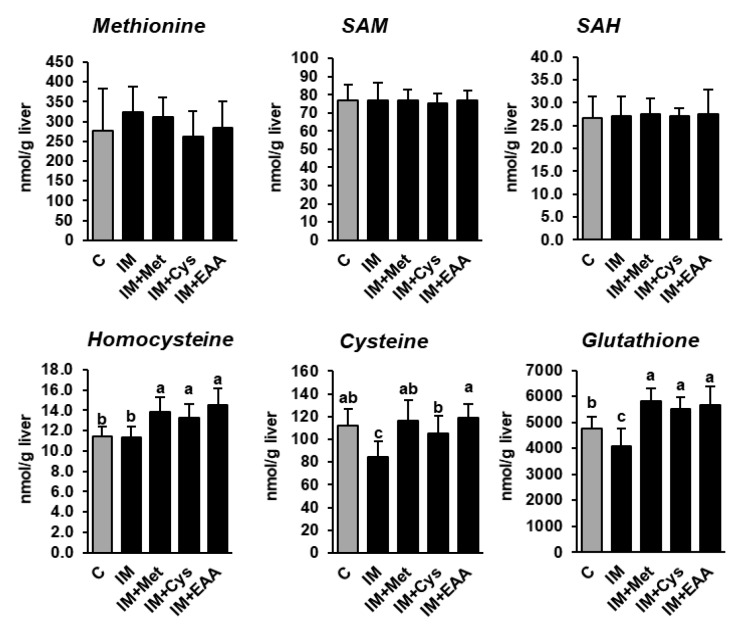
The hepatic concentrations of methionine and its metabolites in obese Zucker rats fed semi-synthetic diets with either casein (C), insect meal (IM), IM with additional methionine (IM + Met), IM with additional cysteine (IM + Cys) or IM with additional essential amino acids (EAA) for 4 weeks. The bars represent means ± SD for *n* = 10 animals per group. Bars without a common letter differ, *p* < 0.05. Abbreviations: SAH, S-adenosylhomocysteine; SAM, S-adenosylmethionine.

The present study shows that the hepatic concentrations of methionine and its demethylation metabolites SAM and SAH did not differ between rats of all groups, and even not between rats of group C and group IM (Table S2), despite the dietary methionine supply being 40% lower in group IM than in group C (Figure 2). In contrast, the hepatic concentrations of cysteine and total glutathione, but not homocysteine, were reduced in group IM compared to in group C (*p* < 0.05, Figure 2), indicating that the transsulfuration of homocysteine was decreased as a consequence of the lower methionine supply in group IM. In fact, it has been recently demonstrated that under conditions of methionine restriction, the re-methylation of methionine from homocysteine is favored over transsulfuration in order to conserve methionine at the expense of cysteine and glutathione [23]. In contrast, the hepatic concentrations of cysteine, glutathione and homocysteine were either higher in groups IM + Met, IM + Cys and IM + EAA than in group C, or did not differ between these groups, indicating that the higher supply of sulfur-containing amino acids attenuated the methionine-sparing effect observed in group IM. However, considering that the inhibitory effect of IM on hepatic lipid concentrations and hepatic lipid synthesis was similarly strong in all IM-fed groups suggests that the alterations in methionine metabolism induced by feeding IM are not mainly responsible for the hepatic lipid-lowering effect of IM.

## 4. Conclusions

The present study clearly shows that the strong liver lipid-lowering effect of IM cannot be substantially reversed by either the adjustment of the dietary methionine concentration between the IM diet and the casein diet, the increased supply of cysteine to the IM diet or the adjustment of the dietary concentrations of EAA between the IM diet and the casein diet. This indicates that a difference in the amino acid composition between IM and casein, a low concentration of methionine in IM and a reduced cysteine synthesis secondary to a decreased methionine availability resulting from feeding IM are not causative for the lipid-lowering effect of IM. A practical consequence of our findings is that the inhibitory effect of IM on hepatic lipid synthesis likely does not disappear by the combined ingestion of IM and other dietary proteins rich in sulfur-containing amino acids or EAA.

Thus, other factors associated with IM, such as biologically active peptides, chitin or any other component of IM may be responsible for its lipid lowering effect. While biologically active peptide sequences with lipid-lowering actions, which are resistant to hydrolysis in the intestinal tract and enter the circulation in intact form, have been identified in various legume proteins [8], their existence in IM remains to be shown. In contrast, chitin, which is an intrinsic constituent of the insect´s exoskeleton and is therefore present in IM, has been demonstrated to exert fat-binding properties in the intestines of men and pigs [34,35] and causes lipid-lowering effects [36]. In the IM diet, chitin made up approximately 3.4%. Thus, future studies, in which the IM is treated with a chitinase before inclusion into the experimental diet, may provide an answer on the contribution of chitin to the lipid-lowering effects of IM. With regard to any other component of IM, steroid hormone-like structures, such as ecdysteroids, may be relevant. Ecdysteroids, from which 20-hydroxyecdysone is the predominant representative in *Tenebrio molitor* larvae [37], are insect hormones regulating molting (ecdysis) and metamorphosis and have been shown to exhibit pleiotropic actions, such as anti-obesity, hypoglycemic and even protein-anabolic effects [38,39,40]. Future studies are warranted to examine the potential role of ecdysteroids in the lipid-lowering effect of IM.

## Figures and Tables

**Table 1:** The composition and nutrient and energy contents of the experimental diets.

	C	IM	IM+Met	IM+Cys	IM+EAA
Components (g/kg)					
Corn starch	530	530	530	530	530
Casein	200	-	-	-	-
Insect meal	-	262	262	262	262
Sucrose	100	100	100	100	100
Soybean oil	50	-	-	-	-
Safflower oil	-	19.2	19.2	19.2	19.2
Linseed oil	-	3.8	3.8	3.8	3.8
Cellulose	56.5	21.9	21.9	20.9	20.8
Mineral mix ^1^	35	35	35	35	35
Vitamin mix ^2^	10	10	10	10	10
TiO2	5	5	5	5	5
L-Cysteine (76.9%)	1.2	-	-	3.3	-
DL-Methionine (99%)	-	0.9	3.1	0.9	3.1
L-Threonine (98.5%)	0.2	-	-	-	-
L-Valine (96.5%)	0.6	-	-	-	-
L-Leucine (99%)	-	-	-	-	3
L-Isoleucine (99%)	-	-	-	-	0.5
L-Phenylalanine (98.5%)	-	-	-	-	2.1
L-Histidine (99%)	-	-	-	-	0.4
L-Lysine (78%)	-	-	-	-	4.9
L-Arginine (98%)	3.5	-	-	-	-
L-Tryptophan (98%)	-	-	-	-	0.1
L-Glutamic acid (98%)	8.0	12.2	9.9	9.9	-
Analyzed crude nutrient and energy content					
Dry matter (% FM)	87.7	89.2	88.9	88.6	88.5
Crude protein (% DM)	23.1	23.0	22.8	22.9	23.1
Crude fat (% DM)	5.3	5.5	5.5	5.5	5.4
Crude ash (% DM)	3.5	4.4	4.3	4.3	4.3
Crude fiber (% DM)	4.6	4.4	4.5	4.4	4.5
Gross energy (MJ/kg DM)	19.5	19.3	19.3	19.3	19.4

^1^ The mineral mix provided the following per kg diet: calcium, 5 g; potassium, 3.6 g; chloride, 1.57 g; phosphorus, 1.56 g; sodium, 1.02 g; magnesium, 0.51 g; sulphur, 0.3 mg; iron, 35 mg; zinc, 30 mg; manganese, 10 mg; copper, 6 mg; chromium, 1 mg; fluoride, 1 mg; iodate, 0.2 mg; molybdate, 0.15 mg; selenium, 0.15 mg; lithium, 0.10 mg. ^2^ The vitamin mix provided the following per kg diet: all-*trans*-retinol, 1.2 mg; cholecalciferol, 25 µg; menadione sodium bisulfate, 0.75 mg; all-*rac*-α-tocopheryl acetate, 50 mg; thiamine HCl, 5 mg; riboflavin, 6 mg; pyridoxine HCl, 6 mg; cyanocobalamine, 0.025 mg; biotin, 0.2 mg; folic acid, 2 mg; nicotinic acid, 30 mg; choline, 1 g; pantothenic acid, 15 mg. Abbreviations: C, casein; Cys, cysteine; DM, dry matter; EAA, essential amino acids; FM, fresh matter; IM, insect meal; Met, methionine.

**Table 2:** The composition of the insect meal from *Tenebrio molitor* L.

	Concentration
Crude nutrients (% DM)	
Crude protein	77.1
Crude fat	10.4
Crude fiber	10.6
Crude ash	4.4
Chitin	13.1
Gross energy, MJ/kg DM	24.1
Amino acids (g/kg FM)	
Alanine	59.5
Arginine	36.5
Aspartic acid^1^	61.4
Cysteine	5.7
Glutamic acid^2^	84.5
Glycine	37.3
Histidine	18.3
Isoleucine	33.5
Leucine	53.2
Lysine	39.6
Methionine	8.8
Phenylalanine	26.9
Proline	42.3
Serine	30.0
Threonine	29.7
Tryptophan	8.3
Tyrosine	54.1
Valine	46.9
Fatty acids^3^ (g/100 g total fatty acids)	
12:0	0.3
14:0	2.4
16:0	17.6
16:1 *n*-9	0.6
18:0	4.5
18:1 *n*-9	36.1
18:2 *n*-6	36.4
18:3 *n*-3	1.2
20:0	0.1

^1^ Sum of asparagine and aspartic acid. ^2^ Sum of glutamate and glutamic acid. ^3^ Only fatty acids with concentrations > 0.1 g/100 g total fatty acids are shown. Abbreviations: DM, dry matter; FM, fresh matter.

**Table 3:** The analyzed concentrations of amino acids and the total lipid fatty acid composition in the experimental diets.

	C	IM	IM+Met	IM+Cys	IM+EAA
Amino acids (g/kg FM)					
Alanine	5.4	12.4	12.2	12.6	12.2
Arginine	9.9	9.3	9.1	9.5	9.3
Aspartic acid^1^	10.8	13.0	12.5	13.4	13.1
Cysteine	1.5	1.4	1.5	3.6	1.5
Glutamic acid^2^	46.7	31.8	29.8	30.1	20.6
Glycine	3.3	9.2	9.1	9.4	9.0
Histidine	5.1	4.6	4.5	4.7	4.8
Isoleucine	9.7	8.4	8.7	8.4	8.5
Leucine	16.7	13.7	13.2	13.9	15.7
Lysine	14.4	9.6	9.3	9.7	12.8
Methionine	5.6	3.2	5.3	3.2	5.1
Phenylalanine	9.1	6.8	6.4	6.9	8.4
Proline	20.6	11.1	10.5	10.9	10.5
Serine	9.5	7.3	7.0	7.5	7.1
Threonine	8.0	7.2	7.2	7.5	7.3
Tryptophan	2.3	2.1	2.1	2.0	2.1
Tyrosine	10.1	12.7	13.2	13.4	12.6
Valine	12.5	11.4	12.4	11.5	10.9
Fatty acids^3^ (g/100 g total fatty acids)					
12:0	0.1	0.2	0.2	0.2	0.2
14:0	0.1	1.3	1.3	1.3	1.3
16:0	11.2	12.9	12.9	12.8	12.6
16:1 *n*-9	0.1	0.3	0.3	0.3	0.2
18:0	5.3	4.1	4.2	4.2	4.1
18:1 *n*-9	26.9	26.1	26.1	26.1	25.5
18:2 *n*-6	49.9	50.0	49.8	49.9	50.8
18:3 *n*-3	4.7	3.8	3.7	3.8	4.0
20:0	0.4	0.3	0.3	0.3	0.3

^1^ Sum of asparagine and aspartic acid. ^2^ Sum of glutamate and glutamic acid. ^3^ Only fatty acids with concentrations ≥ 0.1 g/100 g total fatty acids are shown. Abbreviations: C, casein; Cys, cysteine; EAA, essential amino acids; FM, fresh matter; IM, insect meal; Met, methionine.

**Table 4:** Performance parameters of obese Zucker rats fed semi-synthetic diets with either casein (C), insect meal (IM), IM with additional methionine (IM + Met), IM with additional cysteine (IM + Cys) or IM with additional essential amino acids (EAA) for 4 weeks.

	C	IM	IM + Met	IM + Cys	IM + EAA	*p*-Value
Initial BW, g	337 ± 46	338 ± 44	338 ± 46	337 ± 41	337 ± 43	1.000
Final BW, g	491 ± 45	488 ± 35	484 ± 56	495 ± 36	480 ± 40	0.945
BW gain, g/d	5.53 ± 0.94	5.35 ± 0.73	5.24 ± 0.98	5.63 ± 0.79	5.11 ± 0.85	0.671
Feed intake, g/d	30.3 ± 2.0	31.2 ± 1.6	30.7 ± 2.6	33.0 ± 1.9	31.7 ± 2.6	0.356
Feed:gain ratio, g/g	5.61 ± 0.87	5.95 ± 0.94	6.03 ± 1.07	5.96 ± 0.76	6.34 ± 1.02	0.552
Liver weight, g/100 g BW	5.78 ± 0.70	5.66 ± 0.49	5.72 ± 0.71	6.01 ± 0.50	6.21 ± 0.75	0.301

Values are means ± SD for *n* = 10 animals per group. Abbreviations: BW, body weight.

## Supplementary Materials

The following are available online at https://www.mdpi.com/2072-6643/12/4/987/s1, Table S1: Primer characteristics used for qPCR. Table S2: Plasma amino acid concentrations of obese Zucker rats fed semi-synthetic diets with either casein (C), insect meal (IM), IM with additional methionine (IM + Met), IM with additional cysteine (IM + Cys) or IM with additional essential amino acids (EAA) for 4 weeks.
