# Supplementary material for: Supplementation of Sulfur-Containing Amino Acids or Essential Amino Acids Does Not Reverse the Hepatic Lipid-Lowering Effect of a Protein-Rich Insect Meal in Obese Zucker Rats
Source: Nutrients. 2020 Apr 2;12(4):987. doi: 10.3390/nu12040987 (PMC7230462; doi:10.3390/nu12040987)
Supplement: Supplementary file 1 [file nutrients-12-00987-s001.pdf]

Table 1. Primer characteristics used for qPCR.

Gene symbol	Primer forward (5` to 3`)<br>Primer reverse (5` to 3`)	PCR product size (bp)	NCBI GeneBank	Slope	Correlation coefficient	Primer efficiency
Reference genes						
Actb	GACCTCTATGCCAACACAGT	154	NM_031144	-3.45	1.00	1.95
	CACCAATCCACACAGAGTAC					
Canx	CCAGATGCAGATCTGAAGAC	175	NM_172008	-3.55	1.00	1.91
	CTGGGTCCTCAATTTCACGT					
Mdh1	CAGACAAAGAAGAGGTTGCC	206	NM_033235	-3.30	1.00	2.01
	CGTCAGGCAGTTTGTATTGG					
Target genes						
Acaca	TACAACGCAGGCATCAGAAG	244	NM_022193	-3.54	1.00	1.92
	TGTGCTGCAGGAAGATTGAC					
Elovl5	ATGAACTGGGTTCCCTGCGG	102	NM_134382	-3.37	1.00	1.98
	GGAAGGGACAGAGGACAGGC					
Elovl6	AAGTTTGAACTGCGGAAGCCG	211	NM_134383	-3.57	1.00	1.91

	CACCTAGTTCGGGTGCTTTGC					
Fads1	CATTTCCAGCACCACGCCAA	188	NM_053445	-3.54	1.00	1.92
	TAGAGAGGCAGCAAGGCTGG					
Fads2	CATCGACCGCAAGGTCTACAAC	139	NM_031344	-3.24	1.00	2.04
	CTTGCCCACGAAATCCAGGTC					
Fasn	AGGTGCTAGAGGCCCTGCTA	281	NM_017332	-3.57	1.00	1.91
	GTGCACAGACACCTTCCCAT					
G6pd	TTGTACCAGGGTGATGCCTTCC	199	NM_017006	-3.56	1.00	1.91
	GCTCACTCTGTTTGCGGATGTC					
Hmgcr	TGGCAGGACGCAACCTCTAC	173	NM_013134	-3.53	0.99	1.92
	GGCAGCAGGTTTCTTGTCGG					
Scd	TGCACCCCCAGACACTTGTAA	94	NM_031841	-3.57	1.00	1.91
	GGATGCATGGAAACGCCATAG					
Sqle	TGTTGTGAACGTGCTGGCTC	80	NM_017136	-3.44	0.99	1.95
	AAGCTTTTCGGAGCTGACGC					

Abbreviations: *Acaca,* acetyl-CoA carboxylase alpha; *Actb,* actin beta; *Canx,* calnexin; *Elovl5,* ELOVL fatty acid elongase 5; *Elovl6,* ELOVL fatty acid elongase 6; *Fads1,* fatty acid desaturase 1; *Fads2,* fatty acid desaturase 2; *Fasn,* fatty acid synthase; *G6pd,* glucose-6-phosphate dehydrogenase; *Hmgcr,* 3-hydroxy-3-methylglutaryl-CoA reductase; *Mdh1,* malate dehydrogenase 1; *Scd,* stearoyl-CoA desaturase; *Sqle,* squalene epoxidase.

Table S2. Plasma amino acid concentrations of obese Zucker rats fed semi-synthetic diets with either casein (C), insect meal (IM), IM with additional methionine (IM+Met), IM with additional cysteine (IM+Cys) or IM with additional essential amino acids (EAA) for 4 weeks.

	C	IM	IM+Met	IM+Cys	IM+EAA
			µmol/L plasma		
Alanine	717 ± 93[b]	829 ± 48[a]	750 ± 98[b]	763 ± 66[ab]	728 ± 74[b]
Arginine	169 ± 19	166 ± 15	162 ± 17	160 ± 20	169 ± 28
Aspartic acid	23.4 ± 2.0[b]	30.7 ± 2.2[a]	30.3 ± 3.5[a]	30.0 ± 3.2[a]	29.8 ± 2.7[a]
Citrulline	77.1 ± 4.7[b]	85.5 ± 6.4[a]	83.8 ± 6.5[a]	82.1 ± 7.4[ab]	78.8 ± 6.8[ab]
Cysteine	138 ± 17	144 ± 9	147 ± 20	154 ± 13	151 ± 9
Glutamic acid	161 ± 27[b]	196 ± 26[a]	195 ± 32[a]	184 ± 21[ab]	175 ± 29[ab]
Glutamin	705 ± 80	665 ± 70	685 ± 60	677 ± 50	705 ± 72
Glycine	70.8 ± 10.2[b]	129 ± 16[a]	140 ± 14[a]	139 ± 15[a]	131 ± 11[a]
Histidine	101 ± 11[a]	77.5 ± 4.5[b]	84.8 ± 10.0[b]	83.0 ± 5.6[b]	78.5 ± 7.6[b]
Leucine	273 ± 39[a]	231 ± 21[b]	224 ± 36[b]	223 ± 14[b]	233 ± 28[b]
Lysine	447 ± 73[a]	338 ± 23[bc]	323 ± 36[c]	330 ± 28[c]	383 ± 79[b]
Methionine	59.4 ± 12.7[b]	61.1 ± 6.8[b]	81.8 ± 17.2[a]	57.4 ± 7.4[b]	74.8 ± 11.3[a]
Ornithine	70.6 ± 12.7[c]	105 ± 14[a]	88.3 ± 12.4[b]	94.2 ± 9.7[ab]	97.9 ± 15.1[ab]
Phenylalanine	93.9 ± 8.8	91.3 ± 4.2	87.2 ± 8.6	88.8 ± 4.0	91.7 ± 7.6
Proline	355 ± 83[a]	228 ± 30[b]	211 ± 45[b]	191 ± 37[bc]	182 ± 38[c]
Serine	176 ± 17[c]	209 ± 15[a]	194 ± 17[b]	190 ± 15[bc]	186 ± 13[bc]
Taurine	358 ± 44[b]	370 ± 66[ab]	402 ± 41[ab]	430 ± 68[a]	421 ± 71[a]
Threonine	254 ± 37	263 ± 24	259 ± 20	249 ± 35	234 ± 35
Tryptophan	103 ± 8[a]	85.6 ± 12.6[b]	85.1 ± 8.9[b]	87.4 ± 4.9[b]	86.5 ± 9.1[b]
Tyrosine	151 ± 34	167 ± 27	160 ± 29	149 ± 25	155 ± 32

Valine	427 ± 58^{a}	382 ± 34^{b}	364 ± 61^{bc}	366 ± 34^{bc}	337 ± 38^{c}

Values are means ± SD for $n = 10$ animals per group. Means without a common superscript letter differ, $P < 0.05$.
